# Global mapping of cancers: The Cancer Genome Atlas and beyond

**DOI:** 10.1002/1878-0261.13056

**Published:** 2021-07-20

**Authors:** Carlo Ganini, Ivano Amelio, Riccardo Bertolo, Pierluigi Bove, Oreste Claudio Buonomo, Eleonora Candi, Chiara Cipriani, Nicola Di Daniele, Hartmut Juhl, Alessandro Mauriello, Carla Marani, John Marshall, Sonia Melino, Paolo Marchetti, Manuela Montanaro, Maria Emanuela Natale, Flavia Novelli, Giampiero Palmieri, Mauro Piacentini, Erino Angelo Rendina, Mario Roselli, Giuseppe Sica, Manfredi Tesauro, Valentina Rovella, Giuseppe Tisone, Yufang Shi, Ying Wang, Gerry Melino

**Affiliations:** ^1^ Department of Experimental Medicine Torvergata Oncoscience Research Centre of Excellence, TOR University of Rome Tor Vergata Italy; ^2^ IDI‐IRCCS Rome Italy; ^3^ San Carlo di Nancy Hospital Rome Italy; ^4^ Indivumed GmbH Hamburg Germany; ^5^ Medstar Georgetown University Hospital Georgetown University Washington DC USA; ^6^ Sant'Andrea Hospital University of Rome Sapienza Italy; ^7^ CAS Key Laboratory of Tissue Microenvironment and Tumor Shanghai Institute of Nutrition and Health Shanghai Institutes for Biological Sciences University of Chinese Academy of Sciences Chinese Academy of Sciences Shanghai China; ^8^ The First Affiliated Hospital of Soochow University and State Key Laboratory of Radiation Medicine and Protection Institutes for Translational Medicine Soochow University China

**Keywords:** artificial intelligence, cancer, molecular signature, omics, whole‐genome sequencing

## Abstract

Cancer genomes have been explored from the early 2000s through massive exome sequencing efforts, leading to the publication of The Cancer Genome Atlas in 2013. Sequencing techniques have been developed alongside this project and have allowed scientists to bypass the limitation of costs for whole‐genome sequencing (WGS) of single specimens by developing more accurate and extensive cancer sequencing projects, such as deep sequencing of whole genomes and transcriptomic analysis. The Pan‐Cancer Analysis of Whole Genomes recently published WGS data from more than 2600 human cancers together with almost 1200 related transcriptomes. The application of WGS on a large database allowed, for the first time in history, a global analysis of features such as molecular signatures, large structural variations and noncoding regions of the genome, as well as the evaluation of RNA alterations in the absence of underlying DNA mutations. The vast amount of data generated still needs to be thoroughly deciphered, and the advent of machine‐learning approaches will be the next step towards the generation of personalized approaches for cancer medicine. The present manuscript wants to give a broad perspective on some of the biological evidence derived from the largest sequencing attempts on human cancers so far, discussing advantages and limitations of this approach and its power in the era of machine learning.

## Graphical abstract

Since the publication of The Cancer Genome Atlas data in 2013, the advances in the sequencing techniques allowed us to study cancer through whole‐genome sequencing and multiomics approaches. The vast amount of data generated still needs to be thoroughly deciphered, and the advent of machine learning approaches will be the next step towards personalized approaches for cancer medicine.

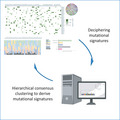

**Abbreviations**
AI: artificial intelligence
ARGO: Accelerating Research in Genomic Oncology
CNAs: copy number alterations
DNS: double‐nucleotide substitutions
ICGC: International Cancer Genome Consortium
InDels: insertions/deletions
PCAWG: Pan‐Cancer Analysis of Whole Genomes
SNS: single‐nucleotide substitutions
SVs: structural variations
TCGA: The Cancer Genome Atlas
TSS: transcription starting site
WGD: whole-genome duplications
WGS: whole-genome sequencing

## 1 Global genomic profiling: from the TCGA to the IGCG‐ARGO projects

As a result of the continuous advances in DNA sequencing techniques and a massive reduction of the associated costs, scientists have been able to move from a classic mechanistic approach, in which a single gene or a set of a few genes were studied to elucidate their roles in cancer development, to global observational analyses. This step has led to the evaluation of the genomic alterations in cancers as a global network of molecular events, generating a huge amount of data from single cancer specimens [1]. Moreover, together with advances in genomics, many other ‘‐omics’ techniques have emerged and have been made available, allowing the generation of multidimensional datasets (genomes, transcriptomes, proteomes, phosphoproteomes, metabolomes) from individuals [2, 3, 4, 5, 6, 7, 8, 9, 10]. This global approach might be regarded as capturing any aspect of the biology of cancer, but also implies a shift in our ability to interpret data and to generalize evidence derived from a single patient to a multitude of individuals with the same disease.

**Fig. 1:**
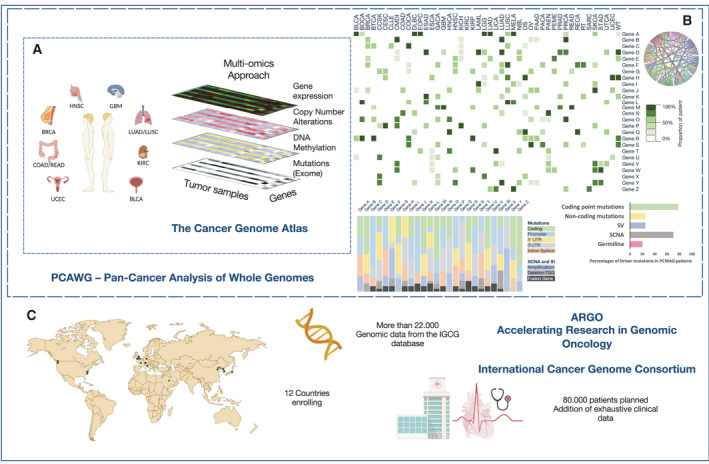
Global cancer genomics approaches. (A) Multiomics approach in The Cancer Genome Atlas (TCGA), the first international project to catalogue the mutational landscape of human cancers. Data from more than 10 000 patients worldwide have been analysed in terms of gene expression, CNAs, DNA methylation and mutations in the coding regions of the genome, providing the mutational landscape of 12 common cancers. (B) The Pan‐Cancer Analysis of Whole Genomes (PGAWG) project analysed more than 2600 whole‐cancer genomes from the International Genome Cancer Consortium (IGCG), building upon previous data from TCGA. Cancer type alteration burden has been evaluated regarding mutations (single base substitutions, double base substitutions, small insertion and deletions), CNAs, SVs and RNA expression (heatmap); genomic alterations have been catalogued according to the site of occurrence, coding region of a gene, regulatory regions as promoter, 5′ or 3′ untranslated regions (5′UTR and 3′UTR) or intron splicing variants, or for CNAs and SVs, providing a specific global profile for each gene alteration; among each class of alterations, driver mutations have been recognized and coding point mutations, together with somatic CNAs (SCNAs), represent the highest number of driver events in cancers (bar chart) [80]. (C) ARGO, Accelerating Research in Genomic Oncology, is the ongoing phase of the global‐scale omics approach of the ICGC, aimed at collecting omics and clinical data from more than 80 000 patients, with the goal to address key biological and clinical questions for each cancer type. This would allow the development of personalized medicine approaches for each cancer patient.

At the early stage of the ‘genomic era’, the accomplishment of the Human Genome Project [11] in sequencing the entire human genome led to the idea that a similar attempt could be applied to cancer genomes. The first ambitious programme with this goal emerged in 2005 – The Cancer Genome Atlas (TCGA). This international multicentre genome sequencing effort took approximatively 8 years to reach completion (Fig. 1A) [12, 13, 14]. TCGA collected exome sequencing data of more than 11 000 cancer samples, characterizing 33 cancer types after an initial exploratory phase on three specific cancer entities (glioblastoma multiforme, lung and ovarian cancer [14, 15, 16]). The amount of data generated, in the order of millions of terabytes, clearly pointed out a crucial issue in the technological support required to process and handle this burden of data. Thus, cloud computing became an essential part of the process, together with the development of more sophisticated algorithms for data interpretation [11, 17, 18, 19].

TCGA analysis of cancer samples mainly focussed on exome sequencing, but complementary approaches such as gene expression profiling and the analysis of copy number alteration (CNAs), single nucleotide polymorphisms, DNA methylation profiling and, to some extent, microRNAs expression were also applied. Only a marginal subset of TCGA cancer samples (< 10%) was used for whole‐genome sequencing (WGS).

The huge amount of data generated allowed scientists to paint a broader picture of the mutational status of cancers and helped in both confirming some of the existing data or in generating new biological hypotheses; for example, on cancer‐related genes [20, 21, 22], apoptotic regulators [23, 24, 25], protein stability [26, 27, 28, 29] and redox regulators [30, 31, 32, 33, 34, 35] or their structural motif [36, 37]. TCGA, however, could not inform on large structural variations (SVs) as noncoding regions were not included in the sequencing.

In recent years, noncoding regions of the human genome expanded as a new focus in the scientific community, with the evidence that alteration of regulatory elements plays an important role in proliferative disorders [38, 39, 40, 41, 42, 43, 44]. Therefore, a further massive sequencing effort to understand cancer from a genomic perspective was launched by the International Cancer Genome Consortium (ICGC). The Pan‐Cancer Analysis of Whole Genomes (PCAWG) started in 2012 with the aim of producing genomic sequences of whole genomes across 38 cancer types, bringing our knowledge on cancer alterations to a more advanced level and allowing the detection of new driver events and the evaluation of large SVs [45] that could not be described by TCGA dataset [46, 47]. In February 2020, the PCAWG published a large part of the results obtained by comparing almost 2700 cancer genomes to their existing normal matching controls, together with almost 1200 transcriptomes (Fig. 1B) [48]. This huge effort allowed scientists to explore, for the first time and in a systematic way, noncoding regions of cancer genomes, and to postulate their role in cancer evolution. The development of mathematical modelling of cancer progression and algorithms also allowed the introduction of ‘molecular timing’ [49] to trace the temporal evolution of a single cancer from a single biopsy.

TCGA and PCWAG still represent the early stage of global mapping of cancer. The two projects still lack a comprehensive collection and analysis of the clinical data of the patients and do not cover proteomics, phosphoproteomics and metabolomics data. The ICGC is therefore now developing the ARGO (Accelerating Research in Genomic Oncology)–IGCG project, aimed at coupling more than 80 000 whole‐cancer genomes to more accurate clinical data from patients (Fig. 1C) [50], but many more projects are starting all around the world [51].

## 2 Mutational signatures from WGS

The massive amount of data generated by the sequencing of whole‐cancer genomes might be used for diverse purposes, mainly dependent on the mathematical approaches used to perform their analysis [52]. A common approach is to describe mutational signatures for specific cancer types. Mutational or structural variations are grouped based on their nature [single‐ or double‐nucleotide substitutions (SNS or DNS), small insertions or deletions (InDels)] [53] and then analysed through cancer types to recognize precise patterns of variations among all the mutations occurring in each cancer sample compared to what happens in the general population. This kind of analysis has been adopted in the context of all the global genomic approaches developed so far (from the TCGA to the PCAWG), as well as in more limited analyses [54, 55, 56, 57, 58, 59, 60].

**Fig. 2:**
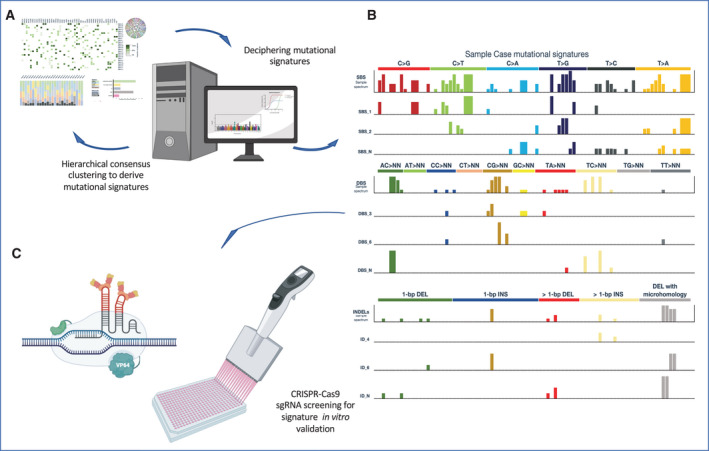
Mutational signatures of cancers. (A) Global genomics data obtained in the Pan‐Cancer Analysis of Whole Genomes (PCAWG) have been processed with fitting algorithm models to recognize mutational signatures in each cancer type. (B) The mutational profile of each cancer type can be dissected in multiple signatures according to the distribution of single base pair (bp) substitutions (SBS), double base substitutions (DBS) or small insertions/deletions (InDels); this approach allows researchers to correlate a specific signature with biological programme alterations in cancers (the APOBEC signature is an example) or with the clinical history of the patient (smoking‐associated signatures) [54]. (C) Signatures can be further investigated in their role in cellular or animal models using CRISPR‐Cas9 technology and single‐guided‐RNA screening platforms.

The most significant attempt applying this approach used data obtained from almost 24 000 cancer samples from different global genomic cancer databases, comprising 2600 samples from the PCAWG. This resulted in the identification of 67 mutational signatures, among which 49 were considered of biological significance (Fig. 2A,B). Many of those could be associated with a specific mechanism through which the tumours arise or progress, as in the case of signatures associated with defective DNA repair [61, 62] or could be linked to clinical aspects and/or therapies, as in the case of signatures associated with platinum‐based compounds [63] or ultraviolet exposure [64].

The comparative algorithms developed to detect mutational signatures were associated with different levels: SNS‐based signatures associated with tobacco habit correlated in a statistically significant way to the tobacco‐associated DNSs or the corresponding InDel signatures, somehow auto‐validating the system [54, 61].

The constant increase of cancer genomic data has also allowed us to refine already known signature, to better separate overlapping ones or to dissect some of them into sub‐signatures [65]. Moreover, mutational signatures could also be associated with endogenous or exogenous exposure to biologically relevant substances linked to cancer development or to known pathogenic processes [66, 67].

With notable exceptions (lung and colorectal cancer), the number of DNS signatures correlated with the number of the SNS. The number of signatures attributable to a cancer type also seems to correlate with the age of diagnosis, suggesting a possible temporal trend in the acquisition of these specific patterns of genomic alterations, introducing the concept that a mutational signature might be active already throughout the cell lineage of the tissue from which the cancer arises, from the fertilized egg onwards.

Although the validity of this approach can generate many biological hypotheses that might better unravel unexplored cancer mechanisms, some of the observations might be biased by the mathematical method applied to derive each signature and therefore need external validation through classical molecular biology approaches. The complexity of the cancer signalling during cancerogenesis and progression [68, 69, 70, 71], as well as the mutational landscape of each signature, could be reproduced in *in vitro* or *in vivo* systems using CRISPR/Cas9 screening libraries (Fig. 2C) [72].

## 3 Application of WGS on large cancer specimen databases

**Fig. 3:**
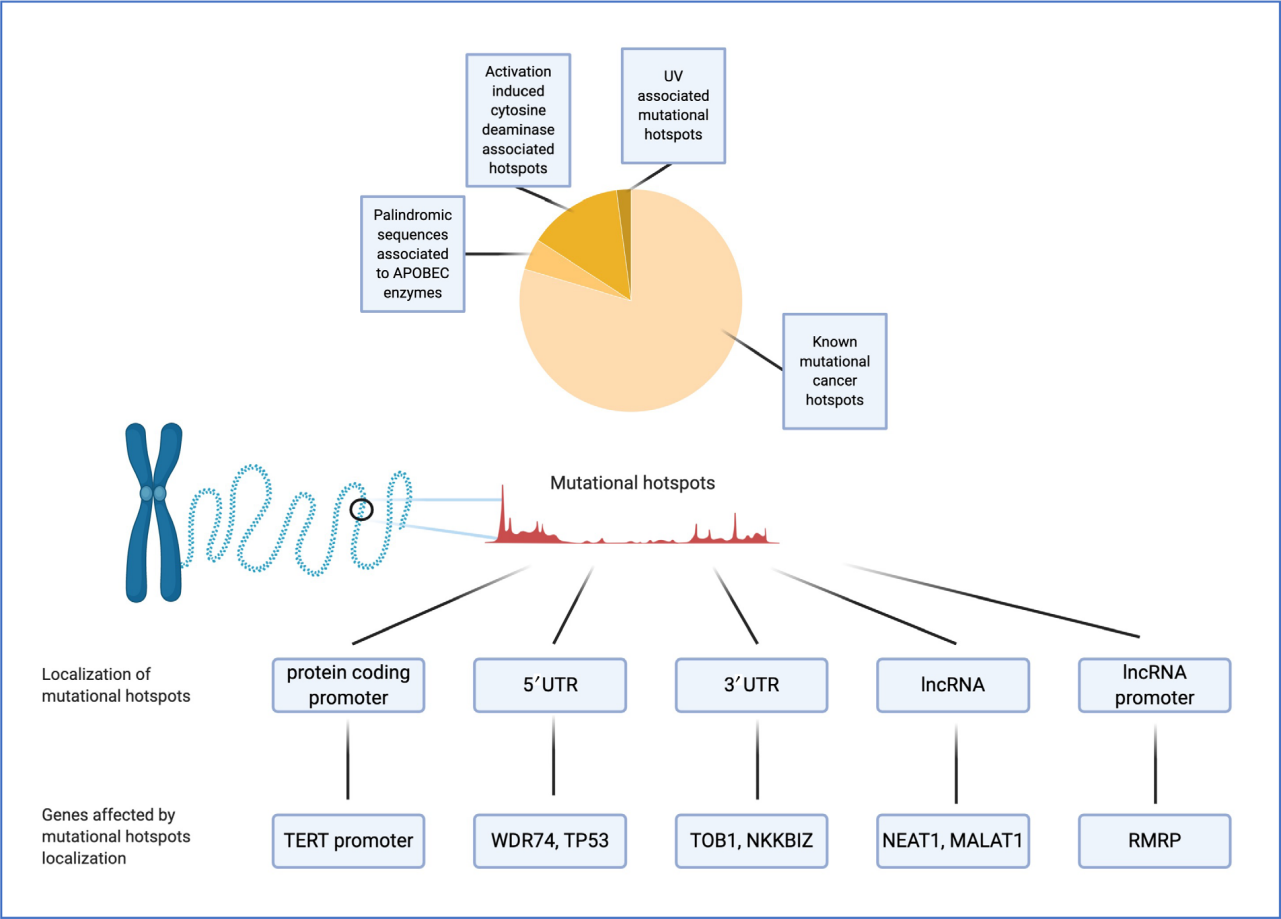
Mutational hotspots of cancers in noncoding regions of the genome. Mutational hotspots in cancer are frequently localized in known mutated genes and can act as drivers. Their frequency in noncoding regions has been recently evaluated [48]. Apart from known hotspots in coding regions, 25% of cancers show clusters of mutations that are localized at the 5′UTR or 3′UTR of genes, as well as on long noncoding RNAs and on their promoters. These hotspots can also be linked to specific signatures, such as UV, activated induced cytidine deaminases and APOBEC enzymes activity [112].

The PCAWG project has been built upon the line traced by TCGA, with more than 2600 cancer samples collected and analysed for WGS, together with transcriptomics data and annotation of somatic small nucleotide variants, CNAs, small InDels, SVs, germline mutations, some retro‐transposition events and mitochondrial DNA defects. Genomic alterations have been ranked based on their recurrence and on their functional consequences, finally developing a clustering methodology to discriminate between potential driver events [46]. The extension of the sequencing to intergenic regions allowed evaluation of the burden of putative driver mutations in noncoding regions: on the pan‐cancer database, 13% of all mutations were represented by driver point‐mutation events in an intergenic region, with 25% of all PCAWG cancers analysed bearing at least one, one‐third of which occurred in the *TERT* promoter, confirming its role in cancer [73, 74, 75, 76, 77, 78, 79]. On the counterpart, 91% of all cancers harboured a somatic driver event in a coding region of a gene (Fig. 3).

The global genomic approach from the PCAWG also raised some interesting perspectives that might be explored in further investigations, since some *bone fide* genetic drivers have not been confirmed with this WGS approach. Not all the mutations in a known cancer‐associated gene (considered a driver gene) are necessarily drivers if analysed through a ‘ranking’ approach, where mutations are ranked not only according to their frequencies but also by their putative functional consequence. This evaluation approach also led to the identification of 181 cancers (from the more than 2600 total analysed cases) without probable driver events (mostly in hepatocellular carcinomas, prostate cancer, medulloblastoma, pancreatic neuroendocrine tumours and renal chromophobe cancers). The heterogeneous nature of this group needs to be further analysed to understand whether possible common features are associated with the lack of putative driver events. This issue also pinpointed a provocative question: is no detection of driver mutations a biological phenomenon yet to be explained, or is it just a technical limitation that could be overcome with alternative approaches?

Besides the general overview on genetic aberrations and on their driver potential, specific cancer types, such as chromophobe renal cancer and neuroendocrine pancreatic tumours, surprisingly showed a higher number of driver mutations if compared to any other cancer type [80].

The WGS approach also explored some possible mechanistic links between catastrophic genomic alteration events, such as kataegis [81]. Kataegis is a mutational event in which a single strand of DNA is hypermutated with clusters of nucleotide substitutions. It was found in more than half of the cancer samples analysed by the PCAWG consortium and was more abundant in lung and bladder cancer, melanoma and sarcomas. Among the driver mutations frequently associated with kataegis foci, some genes are well‐known cancer drivers, such as *CDKN1B*, *EGFR*, *FOXO1*, *MYC*, *SMAD4* and *TP53*, and frequently associated with the APOBEC gene signature [61, 82, 83, 84, 85]. Chromothripsis represents a mutational catastrophe in which hundreds of double‐strand breaks occur in clusters on a few chromosomes [86, 87, 88]. This mutational event was found in almost 600 cases from the PCAWG, frequently in sarcomas, glioblastoma, lung squamous carcinoma melanoma and breast cancer and might explain some of the known pathogenetic features of these diseases [89, 90, 91]. Among the most associated driver genes involved in chromothripsis, *TP53* is prominently represented, reaching statistical significance on the pan‐cancer analysis [10, 85, 92, 93]. *MDM2* and *TERT* amplifications have been associated with chromothripsis in liposarcoma, while *EGFR*, *MDM2* and loss of *CDKN2A* in glioblastoma. As opposed to kataegis, chromothripsis showed correlation to clinical variables being more abundant in women or in late‐onset prostate cancer patients as compared to early onset [80].

## 4 Large structural variations from WGS

Mutations that have been described in the context of cancer signatures, ranging from point mutations to small InDels, are unlikely to affect the genome structurally [94, 95, 96]. Anyway, it is well established that large SVs that endanger the whole structure of chromosomes are at the root of some of the hallmarks of cancers.

Structural variations, represented by large deletions, insertions, translocations or inversions, tend to occur in clusters. Those clusters can be spatially or temporally linked. Most of the time, clusters are both interlinked in space and time, suggesting a possible mechanistic liaison [45].

A WGS approach has the power to detect mutational signatures related to structural variants, and some of the analysis from the PCAWG consortium focussed on this point.

To expand our knowledge on larger SVs, a new classification needs to consolidate classical categories used so far (translocations, inversions, deletions) with the newly identified ones based on the modality of occurrence (the ‘cut and paste’ or the ‘copy and paste’ approach).

A classification of all the possible SVs might be based on the possibility that the inserted segment returns, or not, to the original chromosome. SVs can be classified into chains if they do not return to the original chromosome or into bridges and cycles (leaving a gap or replicating numerous times) if they do return. The way SVs occur can be linked to specific regions of the genome and shed light on the mechanism through which they form: this is the case of the *TERT* regions, where SVs are almost inevitably represented by cycles of templated insertions. This alteration is also associated with SVs affecting tumour suppressor genes, as in the case of the *RB1* gene [45].

Among the classic categories, simple inversions have a relatively low frequency in the PCAWG database and are not associated with copy number gain. A comprehensive evaluation of the complexity of SVs has proved to be challenging; therefore, mathematical approaches like the ones used to highlight mutational signatures have also been used in the context of SVs [97]. At the level of SVs, as in the case of SNS, DNS and InDels signatures, there is a correlation with some described biological processes, as in the case of mutations in the *BRCA2* gene [98], which is associated with small deletions signatures as well as with chromoplexy. This might help in correlating the nature of SVs with a possible mechanism of insurgence and generate more biological hypotheses to explain, from a causative point of view, their insurgence.

## 5 Molecular timing of cancer evolution

Among the possible approaches to analyse global genomics data, an extremely promising one resides in applying bioinformatics algorithms to determine the timing of the evolution of a single cancer. This approach would grant valuable knowledge and allow the development of more accurate and precise early detection techniques [49].

**Fig. 4:**
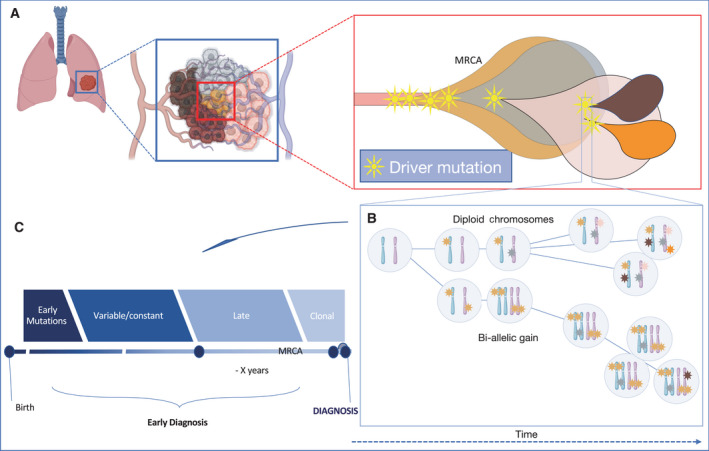
Evolutionary history of cancers, molecular timing and early detection. (A) The mutational history of each cancer can be evaluated from a single biopsy by considering the evolution of tumour heterogeneity. (B) The clonal allelic status of point mutations can be used as a model to classify mutations as preferentially early, variable, constant, late or subclonal. The first two classes of mutations usually harbour driver mutations among many genes, whereas the late and the subclonal classes usually do not contain driver mutations. (C) The classification of mutations according to their type [driver, CNAs, mutational signatures (Sigs)] and their allelic burden allows the reconstruction of a timeline for the development of each tumour [49], potentially extending the time for an early diagnostic approach. MRCA, most recent common ancestor.

Evaluating the number of allelic copies of a mutation of any type might help to discriminate between clonal drivers that are early or late in the evolution of a tumour, whereas a relative ratio among duplicated and nonduplicated mutations would allow determination of the ‘molecular timing’ of a mutation insurgence [99], and the data necessary to develop this kind of approach are obtainable, theoretically, from a single biopsy (Fig. 4A,B) [100], and analysed through a massive sequencing approach, as shown by the data from the PCAWG. Each cancer specimen can be analysed to detect the molecular timing of the mutations found in its genome, and all the information obtained can also be processed through artificial intelligence (AI) approaches to generalize the observations derived from single cases to the comprehensive cancer type to which they belong [99, 100, 101].

One of the first results derived from the PCAWG approach confirmed that many of the most common driver mutations are early clonal. The main example is p53 mutations [102, 103, 104] which are almost exclusively emerging at an early stage of cancer development. Moreover, more than half of all early clonal mutations occur in just nine genes, and these are rarely mutated in later stages or in the subclonal phase (Fig. 4C). This approach can confirm some of the well‐known cancer progression models, as in the case of the *APC*–*KRAS*–*TP53* colon model [105, 106].

Mutations are not the only features of cancer development that can be analysed thorough a timing approach. Signatures, which can also be detected from global genomic sequencing, can be assigned to a molecular time of development. In the PCAWG analysis, signatures associated with exogenous mutagens are invariably found in early development clonal stages, while other signatures tend to accumulate throughout the whole‐cancer evolution, as in the case of the APOBEC signature [49].

The molecular timing analysis considers that the evolution process and the rate of mutations of cancer follow a nonlinear kinetics. This kind of approach allows us to develop a probable timeline of cancer evolution and might represent a good opportunity in cancer prevention, especially in the case of malignancies that are not preceded by a known premalignant lesion. In many cancer types, the early mutations seem to proceed tumour diagnosis by many years. A major limitation is that this analysis is based on point mutations, and therefore, it does not consider any other possible genetic aberrations that might represent *per se* fundamental driver events. Moreover, although the analysis of multiple cancer samples allows the development of this kind of temporal retrospective evolution, many single cases fall out of the prediction, underlining the fact that the nature of cancer is far from deterministic.

## 6 RNA dysregulation in cancer

While the TCGA database systematically collected cancer genomic data by sequencing platforms technologies [107], transcriptomic data have been less methodically collected and, in many cases, the corresponding genomes of the transcriptomes analysed have not been sequenced [108]. Alterations of transcriptomes have therefore been difficult to attribute either to intra‐tumour or inter‐tumour heterogeneity or to the underlying altered genomic landscape. RNA alterations can occur independently from DNA mutations, and therefore, an integrated RNA–DNA sequencing approach is mandatory.

One step forward was made in 2017 by the Genotype‐Tissue Expression (GTEx) consortium, which analysed transcriptomes from 54 healthy tissues from more than 1000 donors, paired with the corresponding WGS [109, 110, 111].

The most recent attempts analysing transcriptomes together with WGS in cancer is again embedded in the PCAWG consortium. The WGS approach allowed researchers to link gene expression variations to transcriptionally inactive genomic sites, such as heterochromatin; more than 2500 cancer specimens were analysed, identifying mutational signatures associated with transcriptional alterations. More than 1100 genes have been associated with these signatures, with an increased number of mutations near the transcription starting site (TSS) of major promoters rather than minor or inactive ones. Mutations in the TSS of major promoters do not seem to significantly alter transcription, since it is more prominent in some cases, such as melanoma, but does not seem to have a role in colorectal cancer [112].

Among the promoters which are highly associated with transcriptional alterations, the *TERT* promoter seems to be the most involved in this kind of deregulation [109, 113, 114].

The exact role of RNA alterations has also been evaluated in the context of signalling pathways, more than focussing on a single gene. The NOTCH and the TGF‐β pathways are largely affected by transcriptional alterations, more than other signalling pathways [112, 115]. Moreover, *KRAS* exhibited more RNA alterations rather than DNA mutations in the context of various cancer types (not all). This finding might also have an impact on the prognostic role of *KRAS* in colorectal cancer and might be considered as a more precise biomarker to determine the fate of this group of patients [112, 116].

Although cancer remains a disease governed primarily by DNA alterations, some driver events are directed by perturbations of RNA expression (which can also depend on noncoding RNAs) [117, 118, 119], which are not depending on underlying genomic abnormalities and gene expression alterations but have rather been shown to be far more associated with CNAs rather than on gene mutations. Anyhow, the PCAWG analysis showed that these cases are quite rare [112]. Altered expression of genes was found in more than 700 genes. For some of them (e.g. *TP53*), RNA alterations were associated with more abundant DNA alterations, whereas others, such as *GAS7*, behaved oppositely. In total, 87 cancer samples could not show any detectable DNA that could justify the RNA alteration observed.

## 7 Capturing genomic alterations during cancer evolution

The focus of the most recent global genomic approaches has been on primary cancer samples. A systematic approach involving WGS on metastatic cancer has been attempted by Priestley *et al*. [120]. WGS of more than 2500 metastatic cancers was analysed and matched with the corresponding genetic background from patient‐derived circulating mononucleated cells.

Among the most interesting highlights derived from this study, more than 80% of the samples harboured whole‐genome duplications (WGDs). This finding contrasts highly with the setting of primary tumours from the PCAWG, where just 30% of the analysed samples showed this kind of genomic duplication [49]. Moreover, the report does not find a recurrent mutation in metastasis, somehow confirming that the metastatic process does not derive from a single driver event but is rather governed by a more pervasive programme.

This ambitious work misses information derived from a parallel WGS of the matching primary cancers. To overcome this limitation, the PCAWG database has been analysed in parallel and confirmed a high genomic concordance between primary and metastatic lesions, also showing that the most common mutation in primary cancers is the same found in metastasis, although at a higher prevalence [121] This finding, together with the evidence of more common WGDs, suggested that a hallmark of metastatic progression might be represented by genomic instability [122, 123]. Conversely to previous results, the tumour heterogeneity of metastatic lesions seems to be less important than the one found in primary cancer; this might further corroborate the idea that a founding cancer cell could colonize a metastatic site and be predominant, but also warns about possible technical limitations derived from biopsy techniques.

## 8 Advantages and limitations of current global cancer genomics approaches and introduction of the executable cancer models

After almost two decades of efforts aimed at collecting large numbers of cancer specimens, generating terabytes of information, where have we arrived? A step towards personalized cancer medicine has undoubtedly been made, since global genomics, as well as global omics, approaches to cancer patients are available almost everywhere and quite accessible in terms of costs [124].

The generation of data has stressed the necessity to work in large international cooperative networks, including TCGA and PCAWG. The attention of the scientific community towards big data generation in cancer has means that the two cited sequencing programmes are not the only ones available: many others, also developed and run by private companies, are concurrently ongoing [125, 126].

A global omics approach also has the prerogative to capture unforeseen cancer alterations that might fit in the context of repurposing and drug rediscovery, which might represent a valuable way in which to exploit pharmacological weapons that we already have but that have not been considered for a given disease.

Alongside the improvement of high‐throughput sequencing techniques, AI has also shown impressive steps forward, with the improvement of both the hardware as well as the computational power of machines [127, 128]. Together with the generation of big data from the ‐omics, AI can be used to develop algorithms to detect cancer signatures that might play a role as more accurate and multidimensional cancer biomarkers [2, 129, 130, 131, 132, 133]. Moreover, multiomics signatures can be associated with specific mechanistic modalities of cancer development (e.g. in the case of signatures associated with exposure to exogenous carcinogens) and can suggest unexplored mechanisms that might cause the oncogenic transformation of cells, therefore impacting on cancer prevention, early detection or therapeutic decisions [121, 134, 135, 136, 137, 138, 139, 140, 141].

Some of the algorithms that have been developed can also allow the early diagnosis of cancer; for example, they provide the possibility to evaluate the ‘molecular timing’ of cancer development, allowing us to infer, from a single biopsy, the mutational evolution of cancer, determining a timeline of evolution for each tumour sample. This kind of approach has shown that, in most cancers, the early‐stage mutations can precede cancer diagnosis by many years. The perfecting of this approach might have an impact on the detection of cancer at a very early phase, also in the context of those neoplasms that do not show a proper premalignant lesion [142, 143, 144, 145].

Many limitations are present in this kind of approach. Undoubtedly, one biological limitation is that cancer heterogeneity is difficult to evaluate from single cancer specimens; moreover, cancer evolution can be inferred from an algorithm, but the dynamic nature of cancer cannot be considered from this single‐biopsy‐based approach. Therefore, the development of less‐invasive procedures to generate ‐omics data are extremely valuable, and liquid biopsies might represent one of the possible new breakthroughs that could have an impact on cancer treatment [146, 147, 148, 149, 150, 151, 152, 153].

An extra level of complexity derives from the evidence that, besides tumour heterogeneity, the tissues employed for omics analysis are mixtures of cancer cells in a complex tumour microenvironment. The complex network between cancer cells and the stroma (immune infiltrates, fibroblasts, endothelial cells) has taken the stage in cancer sciences during the last decade, especially focussing on the immune system liaisons with cancer, resulting in major therapeutical breakthrough, such as the use of checkpoint inhibitors [154, 155, 156, 157, 158]. From one side, prediction algorithms can describe the formation of tumour neoantigens and reconstruct their possible steric presentation by the human leukocyte antigen system of a patient. These *in silico* predicted neoantigens can then be used for the formulation of vaccines that are already tested in clinical trials in some cancer entities [159]. Furthermore, RNA‐seq data can now be deconvoluted to understand the cell types present in a cancer specimen, to evaluate their enrichment, but also weigh the heterogeneity of B and T lymphocytes, thanks to the large‐scale application of the sequencing techniques, which are now more available than before [160, 161].

One other limitation to the current approaches is that the focus is on cancer genomics and transcriptomics. By now, the two largest programmes have only produced a limited amount of data from other ‐omics; data from cancer proteomics, phosphoproteomics, metabolomics and so on could be of greater value if they could be integrated with WGS.

Finally, among the most important limitations linked to past efforts on global genomics approaches is the poor amount of clinical data collected. This point is crucial to integrate this approach in the context of personalized medicine. The importance of clinical data in this setting is clear to the scientific community, and international programmes are already recruiting patients to further expand the ‐omics pool of data supported by a more accurate clinical description of the cases, such as in the GENIE (Genomics Evidence Neoplasia Information Exchange) programme [162] or the ICGC–ARGO [50].

## 9 Executable cancer models: successes and challenges

The large amount of genomic and transcriptomic data available so far contribute to our knowledge on cancer development and progression, but this information is not yet ready to be translated into the clinic. The possibility to choose the right treatment for the right patient is still a goal to reach. Nowadays, it is surely right around the corner, but the huge amount of data obtained from a single patient need wise tools to be interpreted and embedded into the decision‐making process of cancer treatment [163, 164].

Artificial intelligence might just help with this issue. The possibility to develop executable cancer models would allow scientists to search among multiple datasets for the discovery of signatures at every level (genomic, transcriptomic, proteomic, clinical data) to detect key features of the biological behaviour of interest [20, 69, 165, 166, 167]. Biological systems can be treated by AI as networks of information that can be programmed, i.e., reconstructed as a matrix of data [168, 169].

Once the network model is created, the AI continues perfecting it by embedding new data, constantly correcting the generating algorithm to fit the new data. Moreover, discrepancies between a dataset confronted with the executable model might suggest experiments that can be used to further ameliorate or refute the initial hypothesis sustained by the model.

**Fig. 5:**
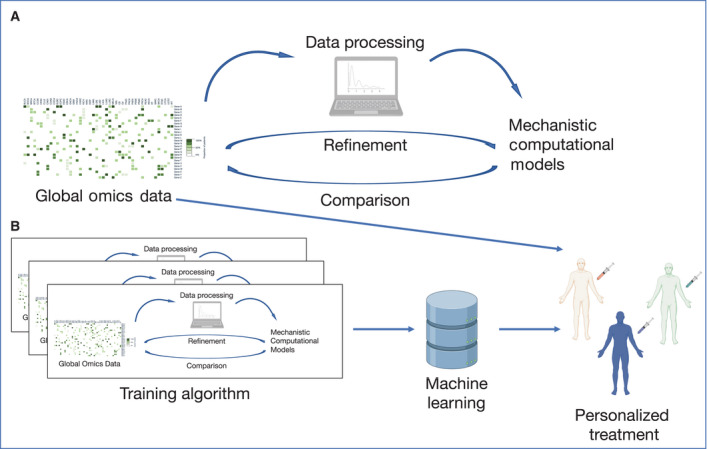
Executable cancer models. (A) Experimental data from a global omics approach can be used as a matrix source for mechanistic computational models that can be continuously processed and refined using data from different cancer types and patients. This will provide data‐based mechanistic hypotheses on each cancer sample. (B) The data obtained through a global omics approach can be further integrated in a machine‐learning system, which is able to refine its ability to highlight mechanistic processes at the root of each cancer sample and can be further integrated with patient‐derived omics and clinical data to develop more precise information of cancer stage and development, ultimately allowing precise personalized medicine interventions.

Due to the extreme plasticity of this kind of approach and to its relative ease of use, AI can overcome one of the limitations that personalized medicine is going through, that is the inability to act according to the evolving patient's response in a timely manner. Nowadays, the maturity of the executable cancer models as such can be easily embedded in a dynamic scenario, such as the one represented by cancer evolution in response to therapies (Fig. 5).

Altogether, the development of AI and the machine‐learning approach should be considered one of the most precious tools for the management, analysis and clinical translation of the endless data obtained from the application of high‐yield sequencing methodologies and proteomics to cancer science and will represent the next major advance in the field, allowing personalized medicine to become an everyday reality.

## Conflict of interest

The authors declare no conflict of interest.

## Author contributions

CG, IA and GM wrote the manuscript. CG prepared the figures. All the other indicated authors (RB, PB, OCB, EC, CC, NDD, HJ, AM, CM, JM, SM, PM, MM, MEN, FN, GP, MP, EAR, MR, GS, MT, VR, GT, YS, YW) made substantial contribution to the conception of the manuscript and critically revised it. All the Authors have approved this submitted version.
